# Transcriptional Differences Guided Discovery and Genetic Identification of Coprogen and Dimerumic Acid Siderophores in *Metarhizium robertsii*

**DOI:** 10.3389/fmicb.2021.783609

**Published:** 2021-11-25

**Authors:** Jinyu Zhang, Peng Zhang, Guohong Zeng, Guangwei Wu, Landa Qi, Guocan Chen, Weiguo Fang, Wen-Bing Yin

**Affiliations:** ^1^State Key Laboratory of Mycology, Institute of Microbiology, Chinese Academy of Sciences, Beijing, China; ^2^Savaid Medical School, University of Chinese Academy of Sciences, Beijing, China; ^3^College of Life Science, Institute of Microbiology, Zhejiang University, Hangzhou, China; ^4^Henan Academy of Science Institute of Biology, Zhengzhou, China

**Keywords:** siderophore, coprogen, dimerumic acid, fungi, biosynthesis, NRPS

## Abstract

Siderophores are small molecular iron chelators and participate in the multiple cellular processes in fungi. In this study, biosynthesis gene clusters of coprogens and dimerumic acids were identified by transcriptional level differences of genes related to iron deficiency conditions in *Metarhizium robertsii*. This leads to the characterization of new coprogen metachelin C (**1**) and five known siderophores metachelin A (**2**), metachelin A-CE (**3**), metachelin B (**4**), dimerumic acid 11-mannoside (**5**), and dimerumic acid (**6**). The structure of metachelin C (**1**) was elucidated by NMR spectroscopy and HR-ESI-MS analysis. Genetic deletions of *mrsidA*, and *mrsidD* abolished the production of compounds **1**–**6** that implied their involvement in the biosynthesis of coprogen and dimerumic acid. Interestingly, NRPS gene *mrsidD* is responsible for biosynthesis of both coprogen and dimerumic acid, thus we proposed plausible biosynthetic pathways for the synthesis of coprogen and dimerumic acid siderophores. Therefore, our study provides the genetic basis for understanding the biosynthetic pathway of coprogen and dimerumic acid in *Metarhizium robertsii*.

## Introduction

Iron is an essential element for the growth and development of all living things on earth. It is an important cofactor involved in the most basic metabolic pathways to sustain life functions, such as the citric acid cycle, DNA replication, and amino acid biosynthesis. Specifically, many enzymes involved in the production of secondary metabolites, like P450 oxidoreductases, require iron or iron-containing cofactors to perform their specific function (He and Mishina, 2004; Misslinger et al., 2017; Dietl et al., 2018; Lill, 2020). Microorganisms absorb iron from the environment for their normal growth and reproduction, but the bioavailability of iron is limited due to its easy oxidation into hardly soluble ferric hydroxides. To overcome the apparent lack of iron, microorganisms have evolved elaborate mechanisms to facilitate iron acquisition. An important way is to secrete siderophores to meet their iron demands. Generally, most fungal siderophores are the hydroxamate-type grouped into four structural families: Rhodotorulic acids, coprogens, fusarinines, and ferrichromes (Hider and Kong, 2010; Haas, 2014). Among them, the biosynthetic pathways of fusarinines and ferrichromes had been well studied (Yuan et al., 2001; Eisendle et al., 2003; Schrettl et al., 2004, 2007). Until now, few biosynthetic pathways of coprogens were reported (Oide et al., 2006; Voß et al., 2020; Zhang J. et al., 2021), and the genetics on rhodotorulic acid biosynthesis has not been documented. Dimerumic acid was reported as a natural siderophore with high affinity for ferric ion that had the same composition as coprogens, except lacking an extra N^5^-anhydromevalonyl-N^5^-hydroxy-L-ornithine (AMHO) unit.

*Metarhizium* spp. are cosmopolitan entomopathogenic fungi, which have been used as environmentally friendly alternative to chemical insecticides (Zhao et al., 2016; Zhang X. et al., 2021). Because of its excellent insecticidal activity, *Metarhizium* spp. had been used as a model system for studying insect-fungal interactions (Xu et al., 2016; Guo et al., 2017). Meanwhile, *Metarhizium* spp. are prolific producers of diverse enzymes and secondary metabolites with insecticidal and pharmaceutical activities (Azumi et al., 2008; Kozone et al., 2009; Krasnoff et al., 2012; Wang et al., 2012; Nishi and Sato, 2019). As natural pest control factors, many secondary metabolites have been widely used in pest control on the market or under development (Fang et al., 2007). However, the biosynthetic gene clusters (BGCs) of *M. robertsii* were obviously not proportional to the identified secondary metabolites (Gao et al., 2011). Previous work has shown that the formation of secondary metabolites was activated by epigenetic modification such as histone acetyltransferase deletion (Fan et al., 2017). Meanwhile, numerous studies have shown that silent gene clusters may be activated by changing transcriptional levels of genes under stress conditions (Amos et al., 2017; Zhang et al., 2019; Zhou et al., 2019). Therefore, we proposed a transcriptional regulation way to mine novel active secondary metabolites in *M. robertsii*.

Here, we aim to find the new siderophores by detecting the transcriptional expressions of BGCs in iron-deficient conditions. Three gene clusters closely related to iron deficiency were identified by RT-PCR analysis, two of which were responsible for the biosynthesis of siderophores. At the same time, we discovered and identified six coprogen and dimerumic acid siderophores including one new natural product. Meanwhile, two biosynthetic genes from different BGCs involved in dimerumic acid biosynthesis were verified by gene knockout and secondary metabolite analysis. Because of widespread existence in fungi, this study provides a basis for further research on coprogens and dimerumic acids.

## Materials and Methods

### Strains, Media, and Culture Conditions

*Metarhizium robertsii* ARSEF2575 were obtained from the Agricultural Research Service Collection of Entomopathogenic Fungi (Zhao et al., 2016). The strains utilized in this work are listed in Supplementary Table 1.

SDAY (Sabouraud–dextrose agar supplemented with 1% yeast extract): Glucose, 10.0 g/L; peptone, 2.5 g/L; yeast extract, 2.5 g/L; and agar, 15 g/L.

AMM: Glucose, 10.0 g/L; NaNO_3_, 6.0 g/L; KCl, 0.52 g/L; KH_2_PO_4_, 1.52 g/L; MgSO_4_⋅7H_2_O, 0.52 g/L; and 1 mL of trace element solution per liter, adjust pH = 6.5 using KOH.

AMM-Fe: Glucose, 10.0 g/L; NaNO_3_, 6.0 g/L; KCl, 0.52 g/L; KH_2_PO_4_, 1.52 g/L; MgSO_4_⋅7H_2_O, 0.52 g/L; and 1 mL of trace element solution (without Fe) solution per liter, adjust pH = 6.5 using KOH.

Trace element solution: FeSO_4_⋅7H_2_O, 1.0 g/L; ZnSO_4_⋅7H_2_O, 8.8 g/L; CuSO_4_⋅5H_2_O, 0.4 g/L; MnSO_4_⋅4H_2_O, 0.15 g/L; Na_2_B_4_O_7_⋅10H_2_O, 0.1 g/L; (NH_4_)_6_Mo_7_O_24_⋅4H_2_O, 0.05 g/L.

Trace element solution (without Fe): ZnSO_4_⋅7H_2_O, 8.8 g/L; CuSO_4_⋅5H_2_O, 0.4 g/L; MnSO_4_⋅4H_2_O, 0.15 g/L; Na_2_B_4_O_7_⋅10H_2_O, 0.1 g/L; (NH_4_)_6_Mo_7_O_24_⋅4H_2_O, 0.05 g/L.

### Purification of Compounds

After incubation for 5 days at 25°C on SDAY plates, the conidia of *M. robertsii* ARSEF 2575 were inoculated into AMM-Fe medium with concentration of 10^7^/L for 10 days at 25°C on a rotary shaker at 150 rpm. After culture, the mycelium and fermentation broth were separated, and extracted, respectively, with 5 L ethyl acetate at room temperature. The organic solvent was evaporated under reduced pressure to yield the crude extracts (1.67 g) of fermentation broth. The crude extracts were divided into 4 fractions (Fraction F1 to F4) by non-ionic macroporous resin eluted with methanol-H_2_O in a gradient manner (v/v, 25:75, 50:50, 75:25, 100:0) (Supplementary Figure 1). Further purification of F2 was carried out by a sephadex LH-20 column chromatography eluted with methanol to give 5 fractions named F2.1 to F2.5. F2.2 was subjected to reversed phase C_18_ silica column chromatography eluted with methanol-H_2_O (v/v, 20:80, 30:70, 40:60, 50:50, 60:40, 70:30, 100:0) to give 7 fractions (Fraction F2.2.1 to F2.2.7). The compound **1** (3.9 mg) was obtained from F2.2.4 by semi-preparative HPLC (72:28 CH_3_CN/H_2_O, 2 mL/min). The compound **2** (26 mg) was obtained from F2.2.3 by semi-preparative HPLC (60:40 CH_3_CN/H_2_O, 2 mL/min), while the compounds **3** (3 mg) and **4** (2.3 mg) were obtained from F2.2.6 by semi-preparative HPLC (58:42 CH_3_CN/H_2_O, 2 mL/min). The compound **5** (20 mg) was obtained from F2.2.2 by semi-preparative HPLC (75:25 CH_3_CN/H_2_O, 2 mL/min), while the compound **6** (5 mg) was obtained from F2.4 by semi-preparative HPLC (68:32 CH_3_CN/H_2_O, 2 mL/min).

### Construction of Deletion Cassettes and Transformation of *M. robertsii*

Gene deletion based on homologous recombination was performed as described previously (Xu et al., 2014). The vector Ppk2-bar-GFP-b was used to construct gene deletion plasmids. The resulting plasmids were then transformed into the *Agrobacterium tumefaciens* AGL-1 for fungal transformation. All DNA fragments in this study were cloned using PCR with the FastPfu high-fidelity DNA polymerase (Transgene Biotech, China) on a T100™ Thermal cycler from Bio-Rad. All PCR products were confirmed by sequencing. The mutants were verified by using diagnostic PCR with appropriate primers. The oligonucleotide sequences for PCR primers synthesized by Shanghai Sango Biotech are given in Supplementary Table 2.

### Chemical Analysis Methods and Equipment Overview

Analytical grade solvents were used for extraction and chromatographic separation. Amberlite XAD-16 macroporous resin (Rohmhaas, Rightleder, United States) and octadecyl silane (ODS) (45–70 μm, Merck, Darmstadt, Germany), and sephadex LH-20 column chromatography (GE Healthcare, United States) were used for column chromatography. Analytical HPLC was conducted with a Waters HPLC system (Waters e2695, Waters 2998, Photodiode Array Detector) using an octadecyl silane column chromatography column (YMC-Pack ODS-A, C_18_, 250 × 10 mm, 5 μm, detector: UV) with a flow rate of 1 mL/min. The solvent system used was a linear gradient of 5–100% acetonitrile in water (with 0.01% formic acid) over a period of 20 min. The column was then washed with 100% acetonitrile for 5 min and equilibrated with 5% acetonitrile for 5 min. HPLC separation was performed on semi-preparative HPLC of SSI HPLC instrument (Scientific Systems Inc., Pennsylvania, United States) using an ODS column (YMC-Pack ODS-A, C_18_, 250 × 20 mm, 5 μm, detector: UV) with a flow rate of 2.0 mL/min. Nuclear magnetic resonance (NMR) spectra were recorded on a Bruker Avance-500 spectrometer (Bruker Corporation, Karlsruhe, Germany) at room temperature using TMS as an internal standard. HR-ESI-MS data were conducted with an Agilent Technologies 6520 Accurate-Mass Q-TOF LC/MS spectrometer equipped with an electrospray ionization (ESI) source.

### RNA Preparation and RT-PCR

*Metarhizium robertsii* was cultivated at 25°C in 20 mL AMM medium and AMM-Fe medium for 4 days. The concentration of the spores is about 1 × 10^7^/L in the AMM (+ Fe or −Fe) medium. Total RNA from the mycelium was extracted using a TranZol™ kit (Transgen Biotech, China). RNA quality was checked in a nucleotide analyzer Quawell Q3000 (Quawell, United States). Single strand cDNA was synthesized using the Fast Quant RT Kit (Tiangen Biotech, China) according to the manufacture’s protocol. Reaction conditions were: 95°C for 3 min followed by 30 cycles of 95°C for 20 s, 60°C for 20 s and 72°C for 20 s. This was followed by one cycle of 72°C for 5 min and 4°C forever. The expression level of actin and gDNA were used as control.

## Results

### Secondary Metabolite Analysis and Related Gene Expression Changes Under Iron Deficiency Conditions

**FIGURE 1:**
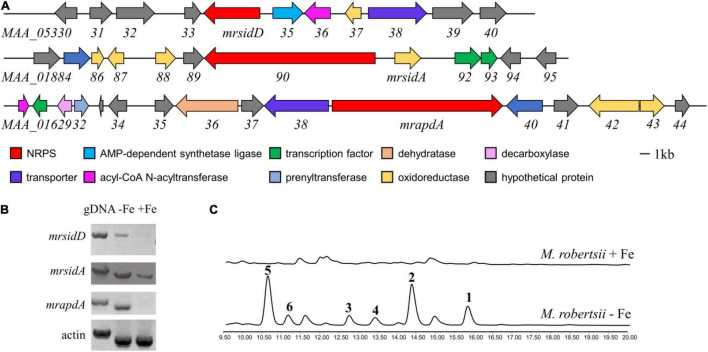
The biosynthetic gene cluster of siderophore and transcriptional analysis of genes related to secondary metabolites under iron deficiency conditions. **(A)** Gene clusters and putative assignments which increased expression at transcriptional level occurred in iron deficiency; **(B)** RT-PCR results of gene expression in the conditions of iron-containing and iron-deficient, the expression levels of actin and gDNA were used as control; **(C)** HPLC traces of *M. robertsii* in the condition of iron-containing and iron-deficient, UV absorption was monitored at 210 nm. 10^4/^mL conidia of *M. robertsii* were point inoculated on AMM medium and AMM-Fe medium and incubated for 10 days at 25°C on a rotary shaker at 150 rpm.

In normal laboratory cultures, many genes involved in key regulatory processes and secondary metabolite biosynthesis are transcriptionally silenced. Iron deficiency upregulated the expression levels of many genes that are not normally expressed or have low expression levels (Blatzer and Latge, 2017). Therefore, it is hoped that more BGCs related to iron concentration can be found through changes in transcription level. In previous studies, the secondary metabolites related to iron concentration in fungi were mostly non-ribosomal peptides, including siderophores (Yin et al., 2013; Kodani et al., 2015). In order to find more genes regulated by iron concentration in *M. robertsii*, we focused on the gene clusters that non-ribosomal polypeptide synthase (NRPS) genes are reside. Because NRPS *mrsidC* (*MAA_01890*) was responsible for the biosynthesis of known compounds ferrichromes (Giuliano Garisto Donzelli et al., 2015), it was not included in the consideration. *M. robertsii* were cultured in iron-containing and iron-deficient media, respectively, and total RNA were extracted. The expression of candidate genes under normal conditions and iron deficiency conditions were detected by RT-PCR analysis, while gDNA and the expression of actin were used as control. The results showed that the expressions of *mrapdA*, *mrsidA*, and *mrsidD* (accession numbers: *mrapdA*, MAA_01639; *mrsidA*, MAA_01891; *mrsidD*, MAA_05334) were significantly up-regulated in iron-deficient condition, but *mrapdA*, and *mrsidD* hardly expressed in iron-containing medium (Figures 1A,B). Transcriptional level results showed that above-mentioned genes were related to iron deficiency. In order to investigate the changes of secondary metabolites under the condition of iron deficiency, *M. robertsii* were cultured in iron-containing medium and iron-deficient medium, respectively. As expected, many differential compounds were produced in the presence of iron deficiency by analyzing the secondary metabolites of *M. robertsii* under different culture conditions (Figure 1C). Employed LC-MS detection showed that the molecular weights of the differential peaks appeared in iron-deficient condition were 485.3, 647.3, 933.4, 1034.5, 1079.5, and 1095.5 [M + H]^+^, respectively. Obviously, iron deficiency does cause significant changes in the secondary metabolites of *M. robertsii*, which may be related to the maintenance of normal physiology and adaptation to adverse environments.

### Characterization of a Series of Mannoside Siderophores in *M. robertsii*

**FIGURE 2:**
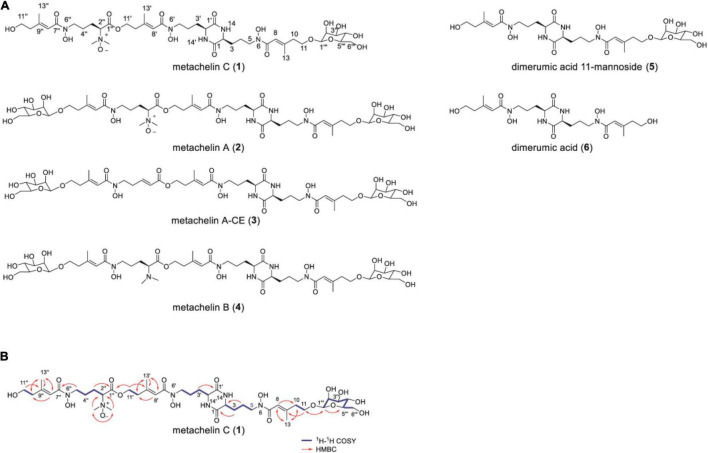
Mannosylated coprogens and dimerumic acids isolated from *M. robertsii*. **(A)** Structures of metachelin C (**1**) and compounds **2**–**6**; **(B)** selected key ^1^H-^1^H COSY, and HMBC correlations of metachelin C (**1**).

To identify the chemical structure of differential compounds produced under iron deficiency conditions, large-scale fermentation of *M. robertsii* was conducted for compound isolation. After culture, the crude extract of *M. robertsii* was subsequently subjected to a combination of non-ionic macroporous resin column chromatography, sephadex LH-20 column chromatography, ODS column chromatography, and semipreparative HPLC to yield compounds **1**–**6** (Figure 2A). Careful analysis of the 1D NMR and HR-ESI-MS data of **2** indicated that it shared the same structure of metachelin A, while compounds **3**–**6** were characterized as known compounds metachelin A-CE, metachelin B, dimerumic acid 11-mannoside, and dimerumic acid, respectively (Krasnoff et al., 2014; Supplementary Tables 6–11). Among them, compound **3** was isolated as naturally occurring compound for the first time. For compound **1**, the molecular formula was determined to be C_41_H_68_N_6_O_18_ based on the HR-ESI-MS signal at *m/z* 934.4670 (calcd. 934.4668) [M + H]^+^. Analysis of the 1D and 2D NMR data (Table 1) and HR-ESI-MS data revealed that metachelin C (**1**) possessed a similar structure to metachelin A (**2**), except that one mannose group in **1** disappeared which were supported by ^1^H-^1^H COSY correlations of H_2_-11″ to H_2_-10″ and the HMBC correlations of H_2_-1″′ to C-11 (Figure 2B). Hence, the structure of **1** was assigned as shown in Figure 2. Structurally, all the compounds were siderophores, which belonged to two major categories: coprogens and dimerumic acids.

**TABLE 1:** NMR spectroscopic data for metachelin C (**1**) in methanol-*d*_4_.

	Metachelin C (1)	
Position	δ_C_^a^	δ_H_ (mult., *J* in Hz)^b^
1/1′	169.0	
2/2′	54.3	4.04, s (2H)
3/3′	31.0/30.9	1.86, m (4H)
4/4′	22.1	1.77, m (4H)
5/5′	46.9	3.69, m (4H)
7/7″	168.4/168.3	–
8/8″	116.3/116.2	6.36, s (2H)
9/9″	152.0/151.2	–
10/10′	40.1/43.4	2.49, t (5.2, 2H)/2.40, t (6.4, 2H)
11/11″	66.7/59.5	4.07, m (1 H); 3.74, m (1H)/3.74, m (2H)
13/13″	17.7/17.5	2.09, s (6 H)
7′	167.9	–
8′	117.5	6.36, s (1H)
9′	149.0	–
10′	38.7	2.58, t (6.1, 2H)
11′	62.7	4.47, m (1H); 4.42, m (1 H)
13′	17.1	2.09, s (3H)
1″	167.6	–
2″	80.2	4.04, m (1H)
3″	25.1	2.05, m (1H); 1.95, m (1H)
4″	23.0	1.69, m (2H)
5″	46.3	3.75, m (1H); 3.69, m (1H)
15	56.8	3.30, s (3H)
15′	53.7	3.26, s (3H)
1″′	100.2	4.55, s (1H)
2″′	71.1	3.86, d (2.9, 1H)
3″′	73.9	3.46, dd (9.3, 2.9, 1H)
4″′	67.2	3.57, t (9.6, 1H)
5″′	77.0	3.24, m (1H)
6″′	61.5	3.90, dd (11.8, 2.2, 1H); 3.72, m (1H)

*^a^Recorded at 125 MHz.*

*^b^Recorded at 500 MHz.*

### Genetic Identification of Genes Associated With Siderophores

**FIGURE 3:**
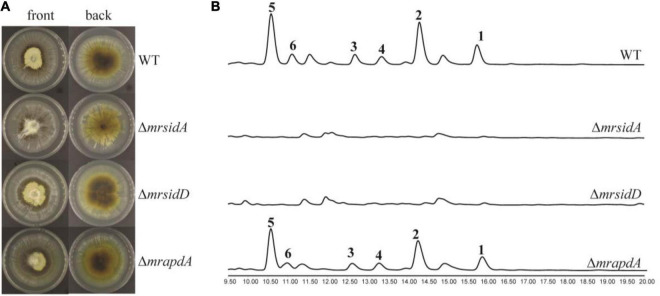
Functional characterization of siderophore biosynthetic genes in *M. robertsii*. **(A)** Growth phenotypes of *M. robertsii* wild-type and mutants. 10^4/^mL conidia of the respective strain were point inoculated on SDAY plates and incubated for 7 days at 25°C; **(B)** HPLC traces of the extracts of wild-type and mutants in iron deficiency condition, UV absorption was monitored at 210 nm. 10^4/^mL conidia of the respective strain were point inoculated on AMM-Fe medium and incubated for 10 days at 25°C on a rotary shaker at 150 rpm.

Under iron deficiency condition, the expressions of *mrsidD*, *mrsidA*, and *mrapdA* were significantly up-regulated expressed, while a series of coprogens and dimerumic acids have been produced. Until now, partial biosynthetic pathways of coprogens in *Metarrhizium* were reported (Giuliano Garisto Donzelli et al., 2015; Donzelli et al., 2017). Dimeric acid was not only reported as a natural siderophore with high affinity for ferric ion, but also had a very good antioxidative effect on hepatocytes (Taira et al., 2002; Yamashiro et al., 2008). To further investigate the correlation of genes with secondary metabolites and assess the function of *mrsidD*, *mrsidA*, and *mrapdA*, the coding region of each gene was deleted by homologous recombination and mutants were verified by the diagnostic PCR (Supplementary Figure 2). Observing the phenotypes of the mutants (Figure 3A), deletion of *mrsidA* showed a significant effect on the growth state of *M. robertsii*, while the other deletion mutants had no significant phenotype changes. Further, the secondary metabolites of wild type (WT) and mutants were extracted and analyzed by HPLC and LC-MS. The results revealed the accumulation of a large amount of coprogens or dimerumic acid in the WT strain but not in Δ*mrsidD*, and Δ*mrsidA* mutants (Figure 3B), while no significant metabolite changes were found in the Δ*mrapdA* mutant compared with the WT. These results indicated that *mrsidD*, *mrsidA* were involved in the biosynthesis of coprogen and dimerumic acid. Bioinformatics analysis of each gene in the two gene clusters according to the genome annotation by BLAST and antiSMASH software (Medema et al., 2011), MrsidD showed high homology with SidD (44.40% ident. 95% coverage) and MrsidA showed high homology with SidA (48.55% ident. 79% coverage), which were all involved in biosynthesis of extracellular siderophores in *Aspergillus fumigatus* (Schrettl et al., 2007). MrsidD was consisted of one complete minimal module and one module lacking the adenylation domain, which was homologous to other fungal proteins (Supplementary Figures 3, 4). Combining the above results, we conclude that the same gene clusters were responsible for the synthesis of mannosylated coprogen and dimerumic acid siderophores.

## Discussion

Iron is an essential trace element for almost all microorganisms, plants and animals on earth. Siderophores have attracted much attention due to their potential role in many biological pathways. In this work, we started at transcription levels, combined with secondary metabolites detection, and eventually identified a series of specific siderophores, mannoside coprogen and dimerumic acid, that were affected by iron concentration. At the same time, BGCs of coprogens and dimerumic acids were identified by combining gene knockout with secondary metabolites detection. In addition, compounds **1**–**5** all contained mannose fragments, but the bioinformatics analysis results showed that the mannose fragment synthesis gene is not in the biosynthesis gene cluster of the siderophores. In other words, there is a broad substrate of glycosyltransferase in the genome that has not been found. If we can find this glycosyltransferase, we can form coprogen and dimerumic acid with more diverse structure and better biological function. Structurally, the coprogen family is one of the widely distributed extracellular siderophore family in fungi (Hof et al., 2009), which consist of three AMHO units, while dimerumic acid was consist of two AMHO units forming a diketopiperazine ring. It is worth mentioned that the two siderophores were synthesized by the same gene clusters. Studies have found that dimerumic acid may be the degradation product of coprogen B through MS/MS and time-course experiments (Silva-Bailao et al., 2014). In addition, from the perspective of gene and compound structure, dimeric acid may also be the precursor in the synthesis of coprogen B leading to the formation of compounds **1**–**4**. Therefore, we proposed the biosynthetic pathways of mannosylated coprogen and dimerumic acid siderophores (Supplementary Figure 5).

It’s also worth noting that the *sidA* gene is responsible for the synthesis of important precursor N^5^ -hydroxy-L-ornithine required for both intracellular and extracellular siderophores. The *sidA* gene in *A. fumigatus* is independent of the siderophores gene cluster, while its homologous gene *mrsidA* exists in *M. robertsii* was located in the intracellular siderophores ferrichromes biosynthesis gene cluster. We carried out homology comparison between the two gene clusters and the known gene clusters of siderophores biosynthesis in coprogen and triacetylfusarinine C. Advanced bioinformatics analysis of the gene cluster indicated that *MAA_05336* had certain homology with GNAT gene *sidF* and *MAA_0533*5 had certain homology with *sidI* at the amino acid level, which both have been shown to be required for the biosynthesis of extracellular siderophore triacetylfusarinine C from *A. fumigatus* (Haas, 2014). Whether there are some interactions between the two siderophores synthesis gene clusters are worth further study.

For both insects and *M. robertsii*, iron is essential for growth and normal physiological activity. After infecting insects, *M. robertsii* releases siderophores to maintain its physiological needs due to the low iron concentration in the insects. The supply of iron is usually limited during a pathogen infection, which is an essential trace element, so adaptation to iron starvation is crucial for virulence (Blatzer and Latge, 2017). *M. robertsii* may compete iron with pests to achieve pest control, which is similar to that of *A. fumigatus* that deficiency of siderophores lead to a reduction in pathogenicity (Weinberg, 1974; Nairz et al., 2018; Khasheii et al., 2021).

In summary, we identified a series of mannosylated coprogen and dimerumic acid siderophores. The structure of one new mannosylated coprogen was elucidated by the comprehensive NMR and HR-ESI-MS analysis. By genetic deletion strategy, we identified the biosynthetic pathway of mannosylated coprogens and dimerumic acids in *M. robertsii*. Furthermore, we proposed the possible biosynthetic pathway for the synthesis of mannosylated coprogen and dimerumic acid siderophores. These two kinds of siderophores are widely present in fungi, so this study provided support for the further application of siderophores in fungi.

## Data Availability Statement

The datasets presented in this study can be found in online repositories. The names of the repository/repositories and accession number(s) can be found below: https://www.ncbi.nlm.nih.gov/, MAA_01639; https://www.ncbi.nlm.nih.gov/, MAA_01891; https://www.ncbi.nlm.nih.gov/, MAA_05334.

## Author Contributions

W-BY, WF, and GC designed and supervised the project. JZ, PZ, GZ, GW, and LQ performed the experiments. W-BY and JZ wrote the manuscript. All authors analyzed the data and discussed the results.

## Conflict of Interest

The authors declare that the research was conducted in the absence of any commercial or financial relationships that could be construed as a potential conflict of interest.

## Publisher’s Note

All claims expressed in this article are solely those of the authors and do not necessarily represent those of their affiliated organizations, or those of the publisher, the editors and the reviewers. Any product that may be evaluated in this article, or claim that may be made by its manufacturer, is not guaranteed or endorsed by the publisher.
