# Supplementary material for: Transcriptional Differences Guided Discovery and Genetic Identification of Coprogen and Dimerumic Acid Siderophores in Metarhizium robertsii
Source: Front Microbiol. 2021 Nov 25;12:783609. doi: 10.3389/fmicb.2021.783609 (PMC8656255; doi:10.3389/fmicb.2021.783609)
Supplement: Supplementary file 1 [file Data_Sheet_1.docx]

Supplementary Material

Transcriptional differences guided discovery and genetic identification of coprogen and dimerumic acid siderophores in *Metarhizium robertsii*

Jinyu Zhang ^1,2†^, Peng Zhang ^1†^, Guohong Zeng ^3^, Guangwei Wu ^1^, Landa Qi ^4^, Guocan Chen ^4^, Weiguo Fang ^3^, Wen-Bing Yin ^1,2*^

^1^ State Key Laboratory of Mycology, Institute of Microbiology, Chinese Academy of Sciences, Beijing, People’s Republic of China

^2^ Savaid Medical School, University of Chinese Academy of Sciences, Beijing, People’s Republic of China

^3^ Institute of Microbiology, College of Life Science, Zhejiang University, Hangzhou, People’s Republic of China

^4^ Henan Academy of Science Institute of Biology, Zhengzhou, People’s Republic of China

^*^ **Correspondence:**

Wen-Bing Yin

E-mail: [yinwb@im.ac.cn](mailto:yinwb@im.ac.cn)

Tel./Fax: +86-10- 64806170

^†^**These authors contributed equally to this work.**

Table of Contents

| Supplementary Tables | S3 |
| --- | --- |
| Supplementary Table 1. Fungal strains used in this study. | S3 |
| Supplementary Table 2. PCR primers used in this study. | S4 |
| Supplementary Table 3. *mrsid1* gene cluster and gene function assignment. | S5 |
| Supplementary Table 4. mr*sid2* gene cluster and gene function assignment. | S6 |
| Supplementary Table 5. *mrapd* gene cluster and gene function assignment. | S7 |
| Supplementary Table 6. HR-ESI-MS data of compounds 1-6. | S8 |
| Supplementary Table 7. NMR data of metachelin B (2). | S9 |
| Supplementary Table 8. NMR data of metachelin A-CE (3). | S10 |
| Supplementary Table 9. NMR data of metachelin B (4). | S11 |
| Supplementary Table 10. NMR data of dimerumic acid 11-mannoside (5). | S12 |
| Supplementary Table 11. NMR data of dimerumic acid (6). | S13 |
| Supplementary Figures | S14 |
| Supplementary Figure 1. Separation process of crude extract of *M. robertsii*. | S14 |
| Supplementary Figure 2. Gene knockout of *mrsidD*, *mrsidA*, and *mrapdA.* | S15 |
| Supplementary Figure 3. The biosynthetic gene clusters of coprogen and dimerumic acid siderophores in *M. robertsii* and the homologs of *sid1*, *sid2* gene cluster in fungi. | S16 |
| Supplementary Figure 4. Domain composition of MrsidD and reported homologous genes. | S17 |
| Supplementary Figure 5. Proposed biosynthetic pathway of metachelin C (1) and dimerumic acid 11-mannoside (5). | S18 |
| Supplementary Figure 6. ^1^H NMR spectrum of metachelin C (1) in methanol-*d*_4_ (500 MHz). | S19 |
| Supplementary Figure 7. ^13^C NMR spectrum of metachelin C (1) in methanol-*d*_4_ (125 MHz). | S19 |
| Supplementary Figure 8. ^1^H-^1^H COSY spectrum of metachelin C (1) in methanol-*d*_4_. | S20 |
| Supplementary Figure 9. HSQC spectrum of metachelin C (1) in methanol-*d*_4_. | S20 |
| Supplementary Figure 10. HMBC spectrum of metachelin C (1) in methanol-*d*_4_. | S21 |
| Supplementary Figure 11. ^1^H NMR spectrum of metachelin A (2) in methanol-*d*_4_ (500 MHz). | S21 |
| Supplementary Figure 12. ^1^H NMR spectrum of metachelin A-CE (3) in methanol-*d*_4_ (500 MHz). | S22 |
| Supplementary Figure 13. ^1^H NMR spectrum of metachelin B (4) in methanol-*d*_4_ (500 MHz). | S22 |
| Supplementary Figure 14. ^1^H NMR spectrum of dimerumic acid 11-mannoside (5) in methanol-*d*_4_ (500 MHz). | S23 |
| Supplementary Figure 15. ^1^H NMR spectrum of dimerumic acid (6) in methanol-*d*_4_ (500 MHz). | S23 |
| Supplementary References | S24 |

**Supplementary Table 1**. Fungal strains used in this study.

| Strains | Description | Reference |
| --- | --- | --- |
| *Metarhizium robertsii* | WT (CGMCC 3.17906) | Zhao et al., 2016 |
| MAA5334 | Δ*MAA_05334*::*bar* | This study |
| MAA1891 | Δ*MAA_01891*::*bar* | This study |
| MAA1639 | Δ*MAA_01639*::*G418* | This study |

**Supplementary Table 2**. PCR primers used in this study.

| Primers | Oligonucleotide sequence (5’-3’) |
| --- | --- |
| bar-REV | GAGACGTACACGGTCGACTC |
| bar-FOR | GAGTCGACCGTGTACGTCTC |
| 1639-5F-F | ggcaaatagaaacgggcaag |
| 1639-5F-R | GCACGGCGGATGTCGGCCGGGCGTCGTTCTGGGCTCATgggtgcgttttttctgagacg |
| 1639-3F-F | GGTACCGCCCCGTCCGGTCCTGCCCGTCACCGactcatcgtgtagtttacttctatcag |
| 1639-3F-R | catttctgtccacacggcc |
| 1639-nest-F | tagggtgggctaacagactg |
| 1639-nest-R | gcattcgaaggagatggagc |
| 1639-RT-F | acctatgctgagcttgatgac |
| 1639-RT-R | gagagtagtctgaccactgtg |
| 1891-5F-F | taaccttggaagcttggcag |
| 1891-5F-R | CACGGCGGATGTCGGCCGGGCGTCGTTCTGGGCTCATggtgtctggctatgtagtctac |
| 1891-3F-F | CTGCCGGTACCGCCCCGTCCGGTCCTGCCCGTCACCGataaccgtcagtatcgacaagt |
| 1891-3F-R | agcgtctgatgctgacacc |
| 1891-nest-F | ccacaattttgtactcggtacaa |
| 1891-nest-R | atgacttggcattagaaacgg |
| 1891-RT-F | agcaacagagctgctgaag |
| 1891-RT-R | cctttgctaaactgcaagcc |
| 5334-5F-F | agcctgtaaccacacctcc |
| 5334-3F-R | tgctaaacttacgagggttgg |
| 5334-RT-F | caagctgatgtcgtcctcg |
| 5334-RT-R | tcgtccaggtcgtcgtgaa |

**Supplementary Table 3**. *mrsid1* gene cluster and gene function assignment.

| **gene** | **putative function** | **homologue strains** | **identity/similarity (%)** | | **BlastP homologue accession** | |
| --- | --- | --- | --- | --- | --- | --- |
| *MAA_01884* | hypothetical protein | *Metarhizium anisopliae* | | 99/99.73 | | KFG82822.1 |
| *MAA_01885* | amidase | *Metarhizium brunneum* ARSEF 3297 | | 100/98.61 | | XP_014546910.1 |
| *MAA_01886* | dioxygenase | *Metarhizium brunneum* ARSEF 3297 | | 100/96.21 | | XP_014546911.1 |
| *MAA_01887* | aldehyde dehydrogenase | *Metarhizium brunneum* ARSEF 3297 | | 100/99.21 | | XP_014546912.1 |
| *MAA_01888* | oxidase | *Metarhizium brunneum* ARSEF 3297 | | 100/98.90 | | XP_014546913.1 |
| *MAA_01889* | hypothetical protein | *Metarhizium brunneum* ARSEF 3297 | | 100/96.87 | | XP_014546914.1 |
| *MAA_01890* | NRPS | *Metarhizium brunneum* ARSEF 3297 | | 100/97.46 | | XP_014546915.1 |
| *MAA_01891* | L-ornithine 5-monooxygenase | *Metarhizium brunneum* ARSEF 3297 | | 100/97.83 | | XP_014546916.1 |
| *MAA_01892* | transcription factor | *Metarhizium brunneum* ARSEF 3297 | | 100/96.71 | | XP_014546917.1 |
| *MAA_01893* | transcription factor | *Metarhizium brunneum* ARSEF 3297 | | 100/97.37 | | XP_014546918.1 |
| *MAA_01894* | hypothetical protein | *Metarhizium brunneum* ARSEF 3297 | | 100/97.07 | | XP_014546919.1 |
| *MAA_01895* | hypothetical protein | *Metarhizium majus* ARSEF 297 | | 100/97.61 | | KIE03923.1 |

**Supplementary Table 4**. *mrsid2* gene cluster and gene function assignment.

| **gene** | **putative function** | **homologue strains** | **identity/similarity (%)** | **BlastP homologue accession** |
| --- | --- | --- | --- | --- |
| *MAA_05330* | hypothetical protein | *Metarhizium brunneum* ARSEF 3297 | 98/98.82 | XP_014549683.1 |
| *MAA_05331* | hypothetical protein | *Metarhizium brunneum* ARSEF 3297 | 100/98.80 | XP_014549682.1 |
| *MAA_05332* | hypothetical protein | *Metarhizium brunneum* ARSEF 3297 | 55/93.68 | XP_014549681.1 |
| *MAA_05333* | hypothetical protein | *Metarhizium brunneum* ARSEF 3297 | 100/99.78 | XP_014549679.1 |
| *MAA_05334* | NRPS | *Metarhizium brunneum* ARSEF 3297 | 100/96.78 | XP_014549678.1 |
| *MAA_05335* | AMP-dependent ligase | *Metarhizium brunneum* ARSEF 3297 | 100/99.31 | XP_014549677.1 |
| *MAA_05336* | acyl-CoA N-acyltransferase | *Metarhizium brunneum* ARSEF 3297 | 100/99.35 | XP_014549676.1 |
| *MAA_05337* | oxidoreductase | *Metarhizium brunneum* ARSEF 3297 | 100/87.41 | XP_014549675.1 |
| *MAA_05338* | transporter | *Metarhizium brunneum* ARSEF 3297 | 100/97.63 | XP_014549674.1 |
| *MAA_05339* | hypothetical protein | *Metarhizium brunneum* ARSEF 3297 | 100/99.16 | XP_014549673.1 |
| *MAA_05340* | hypothetical protein | *Metarhizium brunneum* ARSEF 3297 | 100/97.29 | XP_014549672.1 |

**Supplementary Table 5**. *mrapd* gene cluster and gene function assignment.

| **gene** | **putative function** | **homologue strains** | **identity/similarity (%)** | **BlastP homologue accession** |
| --- | --- | --- | --- | --- |
| *MAA_01629* | acyl-CoA N-acyltransferase | *Metarhizium brunneum* ARSEF 3297 | 100/97.22 |  |
| *MAA_01630* | transcription factor | *Metarhizium robertsii* | 96/100 | EXU98286.1 |
| *MAA_01631* | decarboxylase | *Metarhizium anisopliae* | 100/98.60 | KAF5137730.1 |
| *MAA_01632* | prenyltransferase | *Metarhizium anisopliae* | 100/98.57 | KAF5137729.1 |
| *MAA_01633* | hypothetical protein | *Metarhizium brunneum* | 50/100 | QLI68471. |
| *MAA_01634* | hypothetical protein | *Metarhizium anisopliae* | 95/95.48 | KAF5137728.1 |
| *MAA_01635* | hypothetical protein | *Metarhizium anisopliae* | 100/84.27 | KAF5121148.1 |
| *MAA_01636* | dehydratase | *Metarhizium majus* ARSEF 297 | 100/94.19 | KID95613.1 |
| *MAA_01637* | hypothetical protein | *Metarhizium acridum* CQMa 102 | 100/93.00 | XP_007809219.1 |
| *MAA_01638* | transporter | *Metarhizium majus* ARSEF 297 | 100/95.04 | KID95615.1 |
| *MAA_01639* | peptide synthetase | *Metarhizium brunneum* ARSEF 3297 | 100/96.67 | XP_014539498.1 |
| *MAA_01640* | glycosyltransferase | *Metarhizium acridum* CQMa 102 | 100/90.02 | XP_007809222.1 |
| *MAA_01641* | hypothetical protein | *Metarhizium acridum* | 91/89.49 | KAG8405566.1 |
| *MAA_01642* | reductase | *Metarhizium majus* ARSEF 297 | 100/96.35 | KID95619.1 |
| *MAA_01643* | cytochrome P450 | *Metarhizium robertsii* | 97/99.80 | EXU98273.1 |
| *MAA_01644* | hypothetical protein | *Metarhizium anisopliae* BRIP 53293 | 100/99.67 | KJK77191.1 |

**Supplementary Table 6**.HR-ESI-MS data of compounds **1**-**6**.

|  | **Experiment value** | **Calculated value** |  |
| --- | --- | --- | --- |
| **1** | 933.4870 | 933.4668 | [M + H]^+^ |
| **2** | 1095.5190 | 1095.5197 | [M + H]^+^ |
| **3** | 1034.4672 | 1034.4669 | [M + H]^+^ |
| **4** | 1079.5543 | 1079.5247 | [M + H]^+^ |
| **5** | 647.3138 | 647.3140 | [M + H]^+^ |
| **6** | 485.2607 | 485.2611 | [M + H]^+^ |

**Supplementary Table 7**.NMR data for metachelin A (**2**).

|  | **2** |
| --- | --- |
| position | *δ*_H_ (mult., *J* in Hz) |
| 1/1' |  |
| 2/2' | 4.06 s (2H) |
| 3/3' | 1.87, m (4H) |
| 4/4' | 1.78, m (4H) |
| 5/5' | 3.69, m (4H) |
| 7/7'' |  |
| 8/8'' | 6.36, s (1H) |
| 9/9'' |  |
| 10/10'' | 2.49, m (2H) |
| 11/11'' | 4.08, m (2 H); 3.74, m (2H) |
| 13/13'' | 2.10, s (6 H) |
| 7' |  |
| 8' | 6.36, s (1H) |
| 9' |  |
| 10' | 2.58, t (6.0, 2H) |
| 11' | 4.47, m (1H);4.43 m (1H) |
| 13' | 2.10, s (3H) |
| 1'' |  |
| 2'' | 4.10, m (1H) |
| 3'' | 2.10, m (1H); 1.95, m (1H) |
| 4'' | 1.68, m (2H) |
| 5'' | 3.75 m (1H); 3.69, m (1H) |
| 15 | 3.31, s (3H) |
| 15' | 3.27, s (3H) |
| 1'''/1'''' | 4.56, s (2H) |
| 2'''/2'''' | 3.88, d (2.7, 2H) |
| 3'''/3'''' | 3.47, dd (9.5, 3.1, 2H) |
| 4'''/4'''' | 3.57, t (9.5, 2H) |
| 5'''/5'''' | 3.24, m (2H) |
| 6'''/6'''' | 3.90, dd (11.8, 2.6, 2H);3.72, m (2H) |

**Supplementary Table 8**. NMR data for metachelin A-CE (**3**).

|  | **3** |
| --- | --- |
| position | *δ*_H_ (mult., *J* in Hz) |
| 1/1' |  |
| 2/2' | 4.04 s (2H) |
| 3/3' | 1.86, m (4H) |
| 4/4' | 1.78, m (4H) |
| 5/5' | 3.69, m (4H) |
| 7/7'' |  |
| 8/8'' | 6.35, s (1H)/6.32, s (1H) |
| 9/9'' |  |
| 10/10'' | 2.49, m (2H) |
| 11/11'' | 4.10, m (2 H); 3.74, m (2H) |
| 13/13'' | 2.10, s (6 H)/2.14, s (3H) |
| 7' |  |
| 8' | 6.35, s (1H)/6.32, s (1H) |
| 9' |  |
| 10' | 2.58, t (6.0, 2H) |
| 11' | 4.33, m (2H) |
| 13' | 2.10, s (3H) |
| 1'' |  |
| 2'' | 6.20, brs (1H) |
| 3'' | 6.01, brs (1H) |
| 4'' | 2.69, m (2H) |
| 5'' | 3.75 m (1H); 3.69, m (1H) |
| 1'''/1'''' | 4.563, s (1H)/4.555, s (1H) |
| 2'''/2'''' | 3.88, d (2.7, 2H) |
| 3'''/3'''' | 3.47, m (2H) |
| 4'''/4'''' | 3.57, m (2H) |
| 5'''/5'''' | 3.24, m (2H) |
| 6'''/6'''' | 3.90, mm (2H); 3.72, m (2H) |

**Supplementary Table 9**. NMR data for metachelin A (**4**).

| position | **4** |
| --- | --- |
|  | *δ*_H_ (mult., *J* in Hz) |
| 1/1' |  |
| 2/2' | 4.04 brs (2H) |
| 3/3' | 1.87, m (4H) |
| 4/4' | 1.78, m (4H) |
| 5/5' | 3.69, m (4H) |
| 7/7'' |  |
| 8/8'' | 6.35, s (2H) |
| 9/9'' |  |
| 10/10'' | 2.49, m (4H) |
| 11/11'' | 4.10, m (2 H); 3.74, m (2H) |
| 13/13'' | 2.10, s (6 H) |
| 7' |  |
| 8' | 6.35, s (1H) |
| 9' |  |
| 10' | 2.56, t (6.0, 2H) |
| 11' | 4.36, t (6.3, 2H) |
| 13' | 2.10, s (3H) |
| 1'' |  |
| 2'' | 3.39, m (1H) |
| 3'' | 1.95, m (2H) |
| 4'' | 1.71, m (2H) |
| 5'' | 3.69, m (2H) |
| 15 | 2.46, s (3H) |
| 15' | 2.46, s (3H) |
| 1'''/1'''' | 4.56, s (2H) |
| 2'''/2'''' | 3.87, d (3.0, 2H) |
| 3'''/3'''' | 3.47, dd (9.5, 3.1, 2H) |
| 4'''/4'''' | 3.57, t (9.5, 2H) |
| 5'''/5'''' | 3.24, m (2H) |
| 6'''/6'''' | 3.90, dd (11.8, 2.1, 2H); 3.72, m (2H) |

**Supplementary Table 10**.NMR data for dimerumic acid monomannoside (**5**).

|  | **5** |
| --- | --- |
| position | *δ*_H_ (mult., *J* in Hz) |
| 1/1' |  |
| 2/2' | 4.18 brs (2H) |
| 3/3' | 1.88, m (2H);1.81 m (2H) |
| 4/4' | 1.75, m (2H);1.70, m (2H) |
| 5/5' | 3.70, m (4H) |
| 7 |  |
| 8 | 6.11, s (1H) |
| 9 |  |
| 10 | 2.49, m (2H) |
| 11 | 4.04, m (1 H); 3.85, m (1H) |
| 13 | 1.98, s (6 H) |
| 7' |  |
| 8' | 6.20, s (1H) |
| 9' |  |
| 10' | 2.41, m (2H) |
| 11' | 3.77, m (2H) |
| 13' | 1.98, s (3H) |
| 1'' | 4.68, s (1H) |
| 2'' | 3.96, m (1H) |
| 3'' | 3.63, m (1H) |
| 4'' | 3.56, t (7.3, 1H) |
| 5'' | 3.37, m (1H) |
| 6'' | 3.92, dd (12.2, 1.65, 1H); 3.71, m (1H) |

**Supplementary Table 11**. NMR data for dimerumic acid (**6**).

|  | **6** |
| --- | --- |
| position | *δ*_H_ (mult., *J* in Hz) |
| 1/1' |  |
| 2/2' | 4.18 brs (2H) |
| 3/3' | 1.87, m (2H);1.80 m (2H) |
| 4/4' | 1.77, m (2H);1.70, m (2H) |
| 5/5' | 3.70, m (4H) |
| 7 |  |
| 8/8' | 6.19, s (2H) |
| 9/9' |  |
| 10/10' | 2.40, m (4H) |
| 11/11' | 3.76, m (4 H) |
| 13/13' | 1.97, s (6 H) |

**Supplementary Figure 1.** Separation process of crude extract of *M. robertsii*.

**
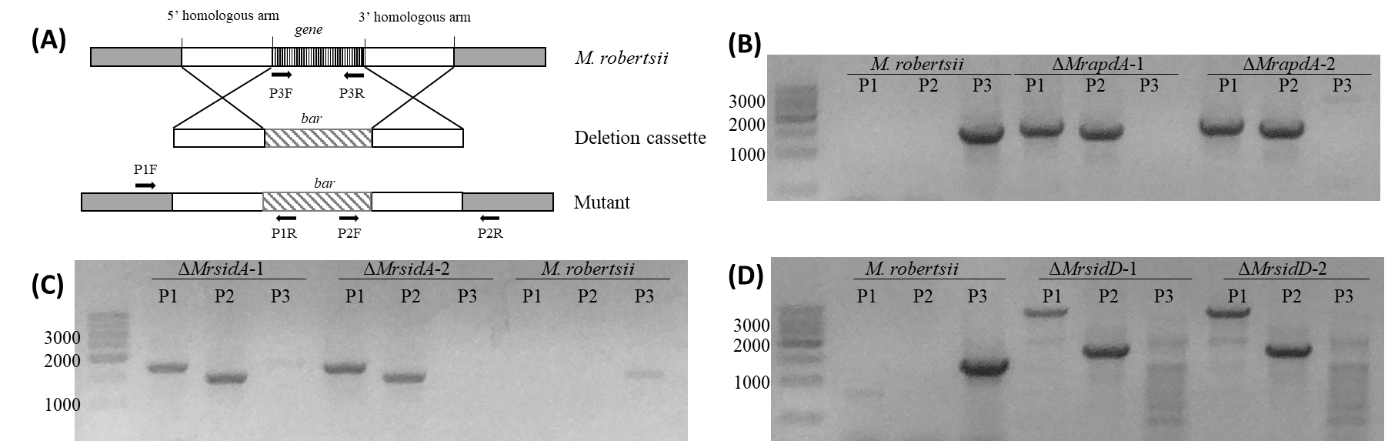
**

**Supplementary Figure 2.** Gene knockout of *mrsidD*, *mrsidA*, and *mrapdA*. (A) Schematic illustration for gene disruption. Three pairs of primers including P1F/R, P2F/R, and P3F/R were used for transformant screening. (B) Transformant screening of knockout *mrapdA*; (C) Transformant screening of knockout *mrsidA*; (D) Transformant screening of knockout *mrsidD*.


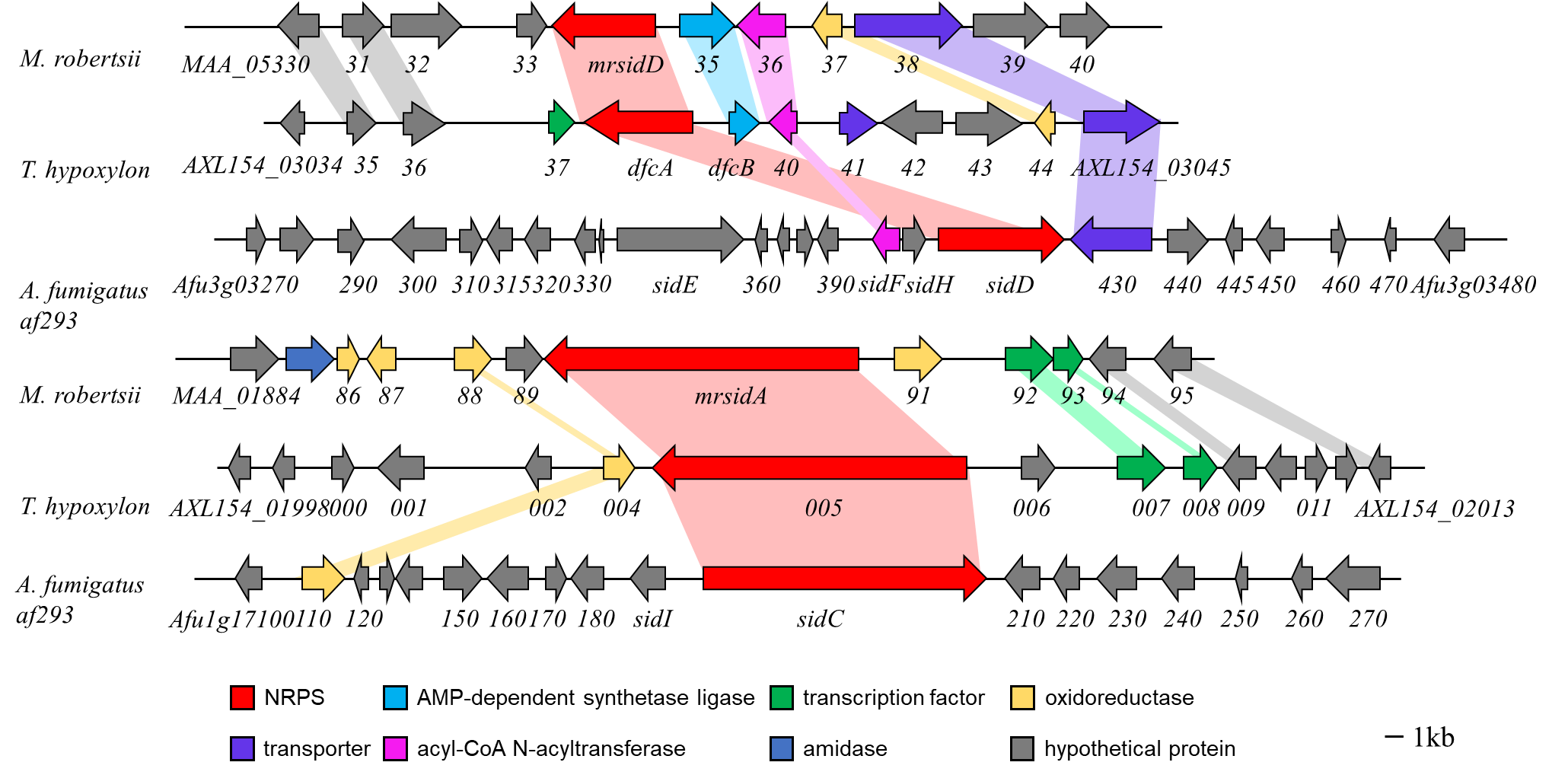


**Supplementary Figure 3.** The biosynthetic gene clusters of coprogen and dimerumic acid siderophores in *M. robertsii* and the homologs of *sid1*, *sid2* gene cluster in fungi. Homologs clusters were found in *A. fumigatus af293*, *T. hypoxylon* (Zhang et al., 2021).

**
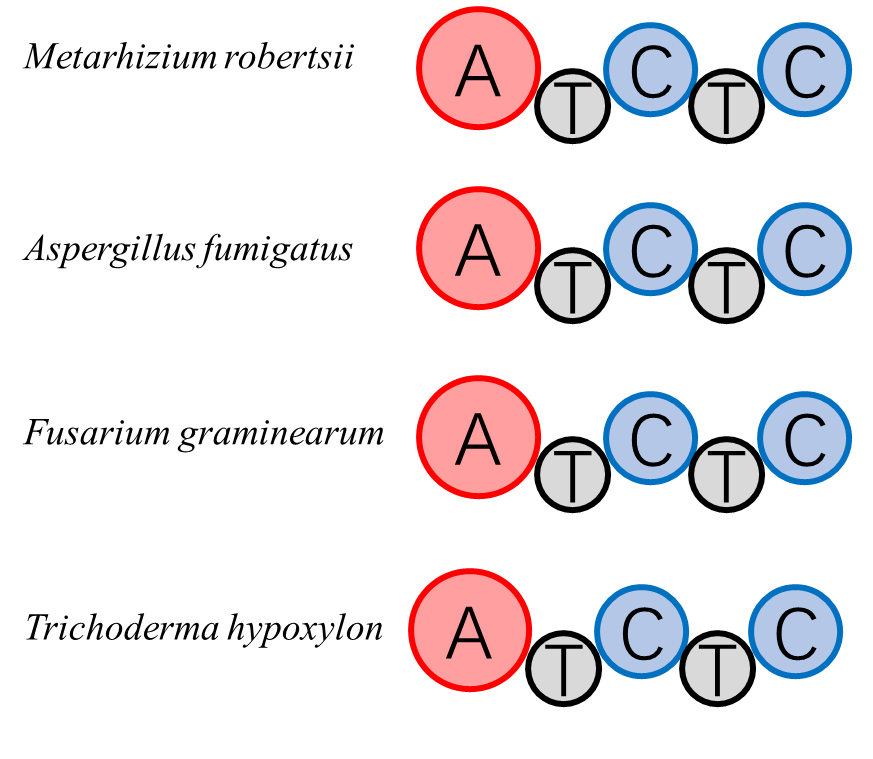
**

**Supplementary Figure 4.** Domain composition of MrsidD and reported homologous genes. *Aspergillus fumigatus* SidD: (accession numbers: Q4WF53.1); *Fusarium graminearum* Nps6 (GenBank: EYB32861.1); *Trichoderma hypoxylon* DfcA: (Zhang et al., 2021).


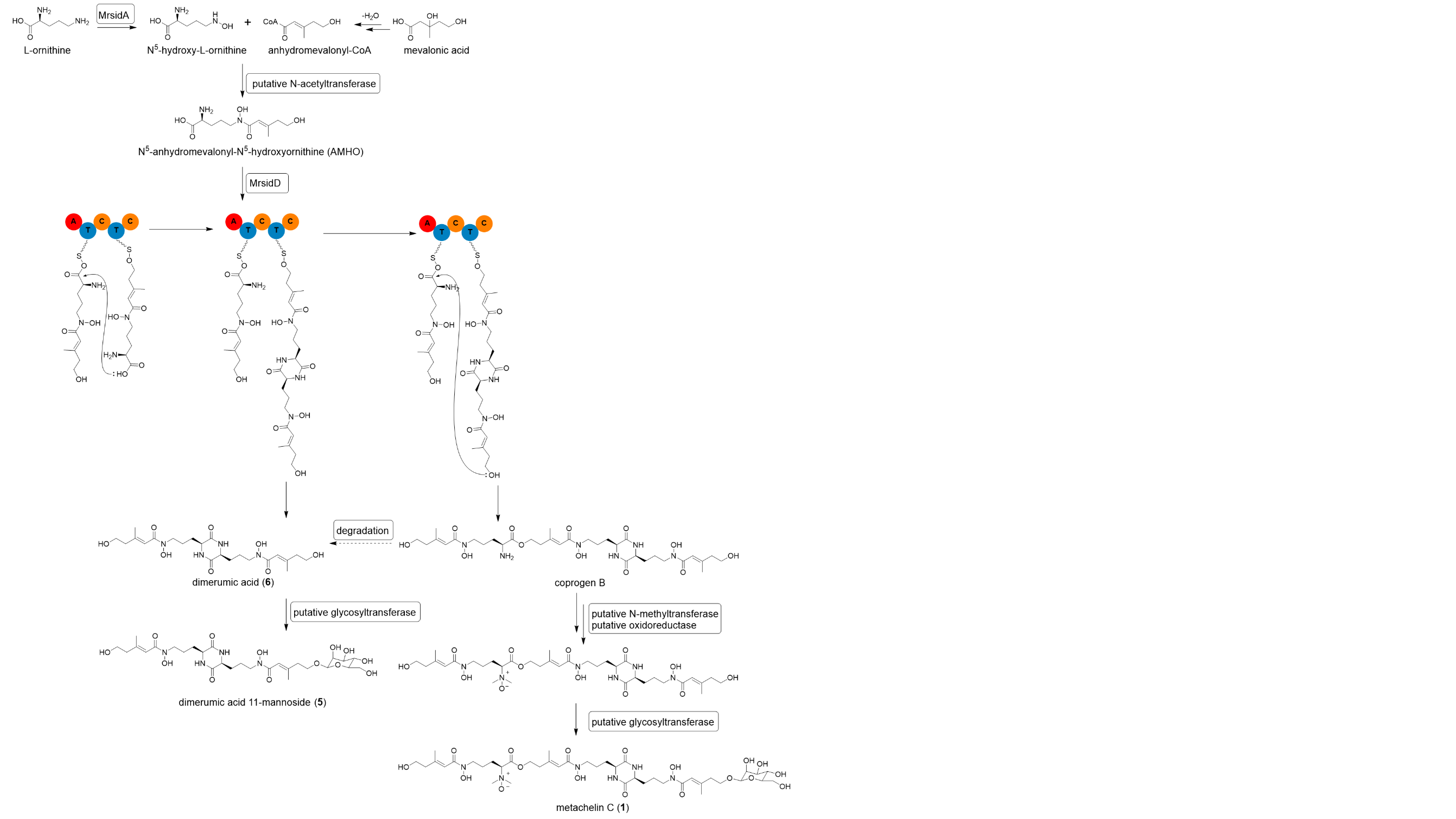


**Supplementary Figure 5.** Proposed biosynthetic pathway of metachelin C (**1**) and dimerumic acid 11-mannoside (**5**).

**Supplementary Figure 6.** ^1^H NMR spectrum of metachelin C (**1**) in methanol-*d*_4_ (500 MHz).

**Supplementary Figure 7.** ^13^C NMR spectrum of metachelin C (**1**) in methanol-*d*_4_ (125 MHz).

**Supplementary Figure 8.** ^1^H-^1^H COSY spectrum of metachelin C (**1**) in methanol-*d*_4_.

**Supplementary Figure 9.** HSQC spectrum of metachelin C (**1**) in methanol-*d*_4_.

**Supplementary Figure 10.** HMBC spectrum of metachelin C (**1**) in methanol-*d*_4_.

**Supplementary Figure 11.** ^1^H NMR spectrum of metachelin A (**2**) in methanol-*d*_4_ (500 MHz).

**Supplementary Figure 12.** ^1^H NMR spectrum of metachelin A-CE (**3**) in methanol-*d*_4_ (500 MHz).

**Supplementary Figure 13.** ^1^H NMR spectrum of metachelin B (**4**) in methanol-*d*_4_ (500 MHz).

**Supplementary Figure 14.** ^1^H NMR spectrum of metachelin A-CE (**5**) in methanol-*d*_4_ (500 MHz).

**Supplementary Figure 15.** ^1^H NMR spectrum of dimerumic acid (**6**) in methanol-*d*_4_ (500 MHz).

**Supplementary References:**

Zhang, J., Qi, L., Chen, G., and Yin, W.B. (2021a). Discovery and genetic identification of amphiphilic coprogen siderophores from *Trichoderm hypoxylon*. *Appl Microbiol Biotechnol* 105(7)**,** 2831-2839. doi: 10.1007/s00253-021-11245-7.

Zhao, H., Lovett, B., and Fang, W. (2016). Genetically Engineering Entomopathogenic Fungi. Adv Genet 94, 137-163. doi: 10.1016/bs.adgen.2015.11.001.
